# Modeling overdispersed longitudinal binary data using a combined beta and normal random-effects model

**DOI:** 10.1186/0778-7367-70-7

**Published:** 2012-04-11

**Authors:** Wondwosen Kassahun, Thomas Neyens, Geert Molenberghs, Christel Faes, Geert Verbeke

**Affiliations:** 1 Department of Epidemiology and Biostatistics, Jimma University, Jimma, Ethiopia; 2 I-BioStat, Center for Statistics, Universiteit Hasselt, Diepenbeek, B-3590, Belgium; 3 I-BioStat, Katholieke Universiteit Leuven, Leuven, B-3000, Belgium

**Keywords:** Bernoulli model, Beta-binomial model, Binomial model, Logistic-normal model, Maximum likelihood

## Abstract

**Background:**

In medical and biomedical areas, binary and binomial outcomes are very common. Such data are often collected longitudinally from a given subject repeatedly overtime, which result in clustering of the observations within subjects, leading to correlation, on the one hand. The repeated binary outcomes from a given subject, on the other hand, constitute a binomial outcome, where the prescribed mean-variance relationship is often violated, leading to the so-called overdispersion.

**Methods:**

Two longitudinal binary data sets, collected in south western Ethiopia: the Jimma infant growth study, where the child’s early growth is studied, and the Jimma longitudinal family survey of youth where the adolescent’s school attendance is studied over time, are considered. A new model which combines both overdispersion, and correlation simultaneously, also known as the combined model is applied. In addition, the commonly used methods for binary and binomial data, such as the simple logistic, which accounts neither for the overdispersion nor the correlation, the beta-binomial model, and the logistic-normal model, which accommodate only for the overdispersion, and correlation, respectively, are also considered for comparison purpose. As an alternative estimation technique, a Bayesian implementation of the combined model is also presented.

**Results:**

The combined model results in model improvement in fit, and hence the preferred one, based on likelihood comparison, and DIC criterion. Further, the two estimation approaches result in fairly similar parameter estimates and inferences in both of our case studies. Early initiation of breastfeeding has a protective effect against the risk of overweight in late infancy (p = 0.001), while proportion of overweight seems to be invariant among males and females overtime (p = 0.66). Gender is significantly associated with school attendance, where girls have a lower rate of attendance (p = 0.001) as compared to boys.

**Conclusion:**

We applied a flexible modeling framework to analyze binary and binomial longitudinal data. Instead of accounting for overdispersion, and correlation separately, both can be accommodated simultaneously, by allowing two separate sets of the beta, and the normal random effects at once.

## Background

In medical and biomedical areas, binary and binomial outcomes are very common. The generalized linear model family [1-3] offers, among others, a suitable modeling framework. Such data are often collected repeatedly in time. Let *r*_*ij*_ be a longitudinal binary outcome for subject *i* at the *j*^*th*^ time point, such that each subject has *n*_*i*_ measurements. The sum $Y_{i}=\sum_{j=1}^{n_{i}}r_{\text{ij}}$ follows a binomial distribution. It is well known that, while i.i.d. Bernoulli variables do not contradict the prescribed mean-variance relation, i.i.d. binomial data can, exhibiting extra variability beyond the binomial model, leading to the so-called overdispersion in the latter, in addition to the correlation emanating from the repeated measures nature. In the past, overdispersion and correlation have been handled separately. To deal with overdispersion, the beta-binomial model is a popular and analytically tractable alternative to the binomial model, which accounts for the overdispersion not accommodated for in the binomial model, thereby allowing for a better fit to the observed data [4,5]. On the other hand, correlation is accommodated for by making use of generalized linear mixed models [6-8], which combine the general exponential family models with normally distributed random effects, are attractive for repeated measurements. In this paper, we use a general and flexible framework for such combinations, proposed by Molenberghs et al [9]. These authors focused on likelihood based methods for inference. In this paper, we have tried to present how the new combined model proposed by Molenberghs et al [9], can be implemented in the Bayesian paradigm. In addition, the ability to specify prior distribution will help to incorporate more information in inference, especially for complex models, like the combined model, that attempt to capture overdispersion and clustering using two separate sets of random effects. Further, we considered two real world data sets and analyzed, first in the likelihood context, and then in the Bayesian, which could also be considered as sensitivity analysis.

Two longitudinal binary data sets, collected in south western Ethiopia: the Jimma infant growth study, where the child’s early growth is studied, and the Jimma longitudinal family survey of youth where the adolescent’s school attendance is studied over time, are considered. One of the key indicators of infant growth is Body Mass Index (BMI). Many studies suggest that Breastfeeding status, and socio-economic condition of the parents, among others, are potential risk factors of BMI [10-12]. School attendance among adolescents varies among gender groups in a way that girls are at higher risk of school absenteeism as compared to boys. Moreover, adolescents living in urban areas have have a better school attendance rate, unlike those in the rural setting [13,14].

The paper is organized as follows. Section (Methods) briefly reviews standard methods and presents the model combining the normal and conjugate random effects in Section (Models combining conjugate and normal random effects). Avenues for parameter estimation and ensuing inferences are explored in Section (Estimation), with particular emphasis on so-called partial marginalization and Bayesian estimation. The results for the analysis are presented in Section (Results) followed by discussed in Section (Discussion). Some concluding remarks are taken up in Section (Conclusion).

## Methods

In this section, we preset the two data sets from the Jimma case studies and briefly describe conventional models used for analysis. We start this section with presenting the data followed by a review of the generalized linear model; we also lay out the notation for the rest of the paper. Section (Overdispersion models) focuses on overdispersion in the binary and binomial situations. Section (Models with normal random effects) reviews the mixed model methodology for longitudinal data analysis. Finally, in Section (Models combining conjugate and normal random effects), the combined model is presented in which ideas from the mixed model methodology are combined with ideas on overdispersion.

### The Jimma case studies

Two longitudinal datasets, Jimma Infant Growth Study and Jimma Longitudinal Family Survey of Youth, collected in Southwest Ethiopia are considered.

The Jimma Infant Survival Differential Longitudinal Growth Study is an Ethiopian study, set up to establish risk factors affecting infant survival and to investigate socio-economic, maternal, and infant-rearing factors that contribute most to the child’s early survival. Children born in Jimma, Keffa and Illubabor, located in Southwestern Ethiopia were examined for their first year growth characteristics. At baseline, there were a total of 7969 infants enrolled in the study, whereby 4317, 1494, and 2158 were from rural, urban, and semi-urban areas, respectively. The children were followed-up every two months, until the age of one year. Of special interest in this manuscript is the risk factor for overweight in children. Overweight, among infants, is associated with various risk factors. It is of particular interest to identify these risk factors in early life through weight and height measurements, which helps in prevention and treatment of overweight and obesity to reduce incidence of several adulthood diseases [15]. This outcome is defined by dichotomization of the Body Mass Index (BMI), with a BMI over the 85th percentile for his or her age referring to overweight. The 85th percentile for age- and sex-specific BMI classification of overweight is used based on Center for Disease Control (CDC) recommendation [16]. The question of interest is whether the percentage of overweight infants changes over time, and whether the evolution differs for gender, place of residence (rural, urban and semi-urban), as well as breast feeding behavior. Table 1 gives a summary of the percentage of overweight infants as a function of gender, location and follow-up time (age).

**Table 1:** Jimma Infant Growth Study

**Time**	**Rural**		**Urban**		**Semi-urban**	
	**Female**	**Male**	**Female**	**Male**	**Female**	**Male**
0	11.5	12.2	16.5	14.5	20.3	21.5
2	12.1	12.7	13.4	13.5	20.6	22.4
4	12.1	12.4	12.7	16.4	22.5	20.2
6	13.4	12.3	13.8	14.9	18.3	21.0
8	12.7	11.8	14.9	19.5	20.2	23.1
10	13.4	11.4	14.9	14.9	19.5	22.6
12	13.8	14.1	16.9	16.0	17.6	18.2

Percentage of overweight male and female infants by place of residence for each of the seven follow-up times.

The Jimma Longitudinal Family Survey of Youth (JLFSY) is another Ethiopian study where data were collected from households. The study began in 2005, and was repeated in 2007. More than 90% of the study subjects present at baseline were visited and willing to respond in the second round. The study population is representative of the relatively large town of Jimma, the small towns of Yebu, Serbo, and Sheki, and nearby rural areas. The sample includes 3700 households as well as 700 adolescents. In this paper, the outcome of interest is the adolescents’ current school attendance coded as 0 (not currently attending) or 1 (currently attending). Current school attendance was 90.2% and 91.1% in the first round survey and 93.5% and 92.8% in the second round for male and 3 female adolescents, respectively. The research question is to examine whether or not the percentage of school attendance depends on adolescents involvement in work to support themselves or their families to earn money, whether they are living in urban towns or rural areas, as well as on gender and age.

### Standard generalized linear model

A random variable Y follows an exponential family distribution if the density is of the form

(1) $$f\left(y\right)\equiv f\left(y|\eta ,\phi \right)=\exp\left\{\phi^{-1}\left[y\eta -\psi \left(\eta \right)+c\left(y,\phi \right)\right]\right\}\text{,}$$

for a specific set of unknown parameters η and ϕ, and for known functions *ψ*(·) and c(·, ·). Often, η and ϕ are termed ‘natural parameter’ (or ‘canonical parameter’) and ‘dispersion parameter,’ respectively. For this family, in general, the mean and variance are related [17].

For binary responses, the model of interest is: *Y*  Bernoulli (π). We want to explain variability between outcome values based on covariate values with density function

(2) $$\begin{array}{cc} f(y|\eta ,\phi ) & =\pi^{y}{\left(1-\pi \right)}^{1-y}\\ & =\exp\left[y\ln\left(\frac{\pi }{1-\pi }\right)+\ln\left(1-\pi \right)\right]\text{.} \end{array}$$

The mean is given by μ = π and the variance, var (μ) = π(1 − π) [1].

When collecting a set of data, let *Y*_1_,…, *Y*_*N*_ be a set of independent binary outcomes, and let *x*_1_,…, *x*_*N*_ represent the corresponding *p*-dimensional vectors of covariate values. With a logit link function, $\ln\left(\frac{\pi_{i}}{1-\pi_{i}}\right)={x^{\prime }}_{i}\xi$ is the logistic regression model with ξ a vector of p fixed, unknown regression coefficients.

### Overdispersion models

The standard Bernoulli model assumes that the mean and variance depend on a single parameter. Though a set of i.i.d. Bernoulli data cannot contradict the mean-variance relationship, it may not hold true for data having a hierarchical structure of the form *z*_*i*_ successes out of *n*_*i*_ trials.

For the Jimma infants study, considering i.i.d. Bernoulli data, the sample average probability of success and the sample variance are 0.150 and 0.128, respectively, indicating that the prescribed mean-variance link is maintained. In contrast, in the binomial setting, taking the hierarchical structure into account, the sample average and the sample variances are 0.141 and 2.107, respectively implying that the sample contradicts the mean-variance relationship for these data.

Similar exploratory analyses on the Jimma Longitudinal Family Survey of Youth were undertaken. For the binomial response, taking the two repeated measurements results in sample average probability of success 0.919 and sample variance 0.168 indicating that the results are in line with the prescribed mean-variance relationship which is known to be always true for the Bernoulli case. This may suggest, at first sight, that these data are not prone to exhibit strong overdispersion, even in the hierarchical binomial setting. In addition to the exploratory analysis, we also made tests for overdispersion. The commonly use approach is to compute the ratio of the residual deviance to the residual degrees of freedom which is approximates the overdispersion parameter ($\hat{\phi }$). When the ratio is appreciably larger than 1, overdispersion is said to occur. It is pointed out that this approach could be misleading when *n*_*i*_*p*_*i*_ is not sufficiently large, where *p*_*i*_ is probability of the success event. This is because it is based on asymptotic theory. As a result, a better approach is based on a quasi-binomial model which allows more dispersion than the binomial model [18]. The approximated overdispersion ($\hat{\phi }$ = 2.37) computed as the ratio of the residual deviance to the residual degrees of freedom in the Binomial, and the one estimated in the quasi-binomial model ($\hat{\phi }$=2.47) for the Jimma Infants Growth data are very similar, both suggesting the presence of strong overdispersion. However, similar analysis for the Jimma Family Survey data does not suggest a considerable overdispersion, with values 0.765 and 1.129, approximated by the ratio of the residual deviance to the residual degrees of freedom in the Binomial, and estimated by the quasi-binomial, respectively.

An elegant way to account for overdispersion is through the so-called beta-binomial model, in which the Bernoulli model is combined with a beta distribution [17,19].

### Models with normal random effects

For non-Gaussian data, the well-known generalized linear mixed model, in which the linear predictor contains random effects in addition to the usual fixed effects, is a common choice [6-8]. These random effects are usually assumed to come from a normal distribution. The model can be specified as follows:

Let Y_ij_ be the jth outcome measured for subject *i* = 1,…, *N* , *j* = 1,…, *n*_*i*_ and group the *n*_*i*_ measurements into a vector ***Y***_*i*_. Assume that, in analogy with Section (Standard generalized linear model), conditionally upon q- dimensional random effects ***b***_***i***_  *N* (**0**, *D*), the outcomes *Y*_*ij*_ are independent with densities of the form

(3) $$f_{i}\left(y_{\text{ij}}|b_{i},\xi ,\phi \right)=\exp\left\{\phi^{-1}\left[y_{\text{ij}}\lambda_{\text{ij}}-\psi \left(\lambda_{\text{ij}}\right)+c\left(y_{\text{ij}},\phi \right)\right]\right\}\text{,}$$

with

(4) $$\begin{array}{ll} \eta \left[\psi^{\prime }\left(\lambda_{\text{ij}}\right)\right] & =\eta \left(\mu_{\text{ij}}\right)\\ & =\eta \left[E\left(Y_{\text{ij}}|b_{i},\xi \right)\right]={x^{\prime }}_{\text{ij}}\xi +{z^{\prime }}_{\text{ij}}b_{i}\text{,} \end{array}$$

for a known link function *η*(·), with *x*_*ij*_ and *z*_*ij*_*p*-dimensional and q-dimensional vectors of known covariate values, with *ξ* a *p*-dimensional vector of unknown fixed regression coefficients, and with ϕ a scale (overdispersion) parameter. Finally, let f (***b***_***i***_|*D*) be the density of the N (**0**, D) distribution for the random effects ***b***_***i***_.

Here, the hierarchical approach is needed because we are working with longitudinal data. More precisely, in our model, the natural parameter is written as a linear predictor, a function of both fixed and random effects.

### Models combining conjugate and normal random effects

Combining both the overdispersion effects (Section Overdispersion models) and the normal random effects (Section Models with normal random effects) into the generalized linear model framework, produces the following general family [9]:

(5) $$f_{i}\left(y_{\text{ij}}|b_{i},\xi ,\theta_{\text{ij}},\phi \right)=\exp\left\{\phi^{-1}\left[y_{\text{ij}}\lambda_{\text{ij}}-\psi \left(\lambda_{\text{ij}}\right)\right]+c\left(y_{\text{ij}},\phi \right)\right\}\text{,}$$

with notation similar to the one used in (3), but now with conditional mean

(6) $$E\left(Y_{\text{ij}}|b_{i},\xi ,\theta_{\text{ij}}\right)=\mu_{\text{ij}}^{c}=\theta_{\text{ij}}\kappa_{\text{ij}}\text{,}$$

where the random variable *θ*_*ij*_G_*ij*_ (ϑ_*ij*_, σ_*ij*_), κ_*ij*_ = *g* (*x*_*ij*_*′***ξ** + *z*_*ij*_′ ***b***_***i***_), ϑ_*ij*_ is the mean of *θ*_*ij*_ and *σ*_*ij*_ is the corresponding variance. Finally, as before, *b*_*i*_  N (**0**, D). Write η_*ij*_ = *x*_*ij*_′ ***ξ*** + *z*_*ij*_′ ***b***_***i***_. Unlike in Section (Models with normal random effects), we now have two different notations, *η*_*ij*_ and *λ*_*ij*_ , to refer to the linear predictor and/or the natural parameter. The reason is that λ_*ij*_ encompasses the random variables *θ*_*ij*_ , whereas *η*_*ij*_ refers to the ‘GLMM part’ only. A detailed overview of the model can be found in Molenberghs et al [9].

For the case of binary data, we assume that

(7) $$y_{\text{ij}}\text{Bernoulli}\left(\pi_{\text{ij}}=\theta_{\text{ij}}\kappa_{\text{ij}}\right)\text{,}$$

(8) $$\kappa_{\text{ij}}=\frac{\exp\left({x^{\prime }}_{\text{ij}}\xi +{z^{\prime }}_{\text{ij}}b_{i}\right)}{1+\exp\left({x^{\prime }}_{\text{ij}}\xi +{z^{\prime }}_{\text{ij}}b_{i}\right)}\text{,}$$

where θ_*ij*_  Beta(α, β). Indeed, this model also intuitively seems useful, as overdispersion and correlation due to the data hierarchy can occur simultaneously.

The model is a two-level model with two types of random effects: (a) the *b*_*i*_, to accommodate correlation among repeated measures (and some overdispersion); (b) the *θ*_*ij*_ for additional overdispersion. While (a) turns the model into a two-level model, rather than a one-level one, (b) does not further add a level, because it merely accommodates overdispersion. This is to be compared with a classical generalized linear model, where also overdispersion random effects can be taken into account (e.g., beta in the Bernoulli model to yield the beta-binomial; gamma in the Poisson model to yield the negative binomial; etc.), while keeping the so-resulting models remain one-level models.

Further, because the *θ*_*ij*_ follow a conjugate distribution, they do not have an impact on the shape of the regression function (like the normal random effects in a linear mixed model), hence there is greatly reduced sensitivity to assumptions about the random effects. This is one of the elegant properties of conjugate random effects.

### Estimation

In the likelihood framework, estimation proceeds by integration. The likelihood contribution of subject *i* is

(9) $$\begin{array}{l} f_{i}\left(y_{i}|\vartheta ,D,\vartheta_{i},\Sigma_{i}\right)\\ =\int \prod_{j=1}^{n_{i}}f_{\text{ij}}\left(y_{\text{ij}}|\vartheta ,b_{i},\theta_{i}\right)f\left(b_{i}|D\right)f\left(\theta_{i}|\vartheta_{i}\Sigma_{i}\right)db_{i}d\theta_{i}\text{.} \end{array}$$

From this, the likelihood is given as:

(10) $$\begin{array}{c} L\left(\vartheta ,D,\vartheta ,\Sigma \right)=\prod_{i=1}^{N}f_{i}\left(y_{i}|\vartheta ,D,\vartheta_{i},\Sigma_{i}\right)|\\ =\prod_{i=1}^{N}\int \prod_{}^{n_{}} \end{array}$$

Here, ϑ groups all parameters in the conditional model for *Y*_*i*_. In the binomial case, the expression takes the form:

(11) $$\begin{array}{l} f\left(z_{\text{ij}}|n_{\text{ij}},b_{i}\right)\\ =\sum_{t-0}^{n_{\text{ij}}-z_{\text{ij}}}{\left(-1\right)}^{t}\kappa_{\text{ij}}^{z_{\text{ij}}+t}\frac{n_{\text{ij}}!}{z_{\text{ij}}!t!\left(n_{\text{ij}}-z_{\text{ij}}-t\right)!}\frac{B\left(z_{\text{ij}}+t+\alpha_{j},\beta_{j}\right)}{B\left(\alpha_{j},\beta_{j}\right)} \end{array}$$

with

(12) $$\kappa_{\text{ij}}=\frac{\exp\left({x^{\prime }}_{\text{ij}}\xi +{z^{\prime }}_{\text{ij}}b_{i}\right)}{1+\exp\left({x^{\prime }}_{\text{ij}}\xi +{z^{\prime }}_{\text{ij}}b_{i}\right)}$$

It is straightforward to obtain the fully marginalized probability by numerically integrating over the normal random effects, and using a tool such as the SAS procedure NLMIXED that allows for normal random effects in arbitrary, user-specified models. More details can be found in [9].

As an alternative estimation method, we turn to the Bayesian paradigm, combined with the popular Markov Chain Monte Carlo (MCMC) technique, making analyses of real-world complex data feasible [15]. In the Bayesian approach, prior distributions are assigned to the parameters and the random effects to adjust for parameter uncertainty. Bayesian inference for estimation of parameter θ is based on the posterior distribution, which is proportional to the likelihood multiplied with the prior distribution.

The Jimma longitudinal studies are characterized by clustering, resulting form the repeated measurements, leading to both correlation and overdispersion. When modeling such data, incorporating prior distribution for model parameters, including that of subject and observation specific random effects, will better handle the underlying uncertainties, instead of assuming that they are fixed. With the same model specification as in the likelihood framework, the parameters ξ, b_*i*_, and θ_*ij*_ are taken to be a priori independent, i.e., *p*(*ϑ, D, ϑ*_*i*_*,* Σ_*i*_) = *p*(*ϑ*)*p*(*D*)p(*ϑ*_*i*_)*p*(Σ_*i*_) and the following prior distributions are used:

ξ  N(0,10^−6^), b_*i*_  N(0,τ_*i*_), as also suggested in the literature [15,16], and θ_*ij*_  Beta(α,β), is unimodal and concave, when α > 1, β > 1 [3]. For the hyper parameters τ_*i*_, the inverse-Gaussian prior *IG*(0.001, 0.001), and for α and β, an improper uniform prior is used, as also suggested by Gelman et al [16]. For more information on Bayesian data analysis and MCMC methods see [20,21].

Note that the Beta-binomial distribution is a compound distribution of the binomial and its conjugate beta, which can be used to capture overdispersion in binomial data. Beta-binomial approximates the binomial distribution arbitrarily well when its two non-negative parameters, α and β, determining its shape, are sufficiently larger. If one or both of these parameters are less than 1, then the probability mass function will go to infinity near its boundaries, 0 and 1, and hence not concave. As a result, the mode does not exist, leading to computational problems in MCMC. For this reason, we used the restriction α > 1, β > 1, such that the density is always concave and unimodal whereby it is always finite over the support [0, 1].

Spiegelhalter et al [22] suggest to use the so-called Deviance Information Criterion for model com- parison in Bayesian inference. Assume a probability model P (*y|θ*). The effective number of param- eters with respect to a model with parameter Θ is given by *pD*{*y*, Θ, $\tilde{\theta }$(*y*)} = E_θ|*y*_ [−2 log *p*(*y*|θ)] + 2 log*p*{*y*|^θ^(*y*)}]. We shall usually drop the arguments {*y*, Θ, $\tilde{\theta }$(*y*)} from notation. Generally, we take $\tilde{\theta }$(*y*) = *E*(θ|*y*), the posterior mean of the parameters. For f (*y*) being a fully specified standardizing term that is a function of the data alone, pD, defined as a ‘mean deviance minus the deviance of the means,’ is given by *pD* = *ED*(θ|*y*)] − *D*(*E*[θ|*y*), where *D*(θ) = −2 log *P* (*y*|θ) + 2 log *f* (*y*) is the Bayesian deviance, used as a measure for goodness of fit. The deviance information criterion (DIC), defined as the classical estimate of fit plus twice the effective number of parameters DIC = *D*(*E*[θ|*y*) + 2*pD* = *ED*(θ|*y*)] + *pD* is used for model comparison. According to this criterion, the model with the smallest DIC is to be preferred. *pD* and *DIC* are easily computed using 7 the available MCMC output by taking the posterior mean of the deviance to obtain *ED*(θ|*y*)] and the plug-in estimate of the deviance *D*(*E*[θ|*y*) using the posterior means *E*[θ|*y* of the parameter θ. In non-hierarchical models, *pD* approximates the effective number of parameters to be estimated. However, for hierarchical models, *pD* is a measure of model complexity instead of being merely the number of effective parameters to be estimated. For the best model preferred based on DIC, the important risk factors could be identified looking the credible intervals, considering whether zero is in or outside of the credible interval.

We also attempted to fit the beta-binomial marginal density, although it is not one commonly encountered in software packages like WinBugs, where an observation x_*i*_ contributes a likelihood term L_*i*_. We used the so-called zero trick, a Poi(ϕ) observation of zero has likelihood exp(−ϕ), so if our observed data is a set of 0’s, and ϕ_i_ is set to − log(*L*_*i*_), we would obtain the correct likelihood contribution [23]. This zero trick allows for arbitrary sampling distributions and is particularly suitable when, say, dealing with truncated distributions. However, our case studies showed that this method can be very inefficient and give a very high Monte Carlo error.

In terms of parameter interpretation, we would like to refer back to the beneficial properties that come with the conjugacy property. Indeed, because the θ_*ij*_ follow a conjugate distribution, the interpretation of the parameters is the same as in a classical generalized linear mixed model. Precisely, this means that the effect on the regression parameters only comes from the normal random effects in the linear predictor, a fact well documented. For a review, see, for example, Molenberghs and Verbeke [17].

## Results

### The jimma infant growth study

We will analyze the binary BMI data. The following model is assumed for the mean structure:

*Y*_*ij*_ |b_*i*_  Bernoulli(π_*ij*_), for subject *i* and measurement *j*, and

(13) $$\begin{array}{ll} \log it\left(\pi_{\text{ij}}\right) & =\xi_{0}+b_{0i}+\left(b_{1i}+\xi_{1}\right)T_{\text{ij}}+\xi_{2}G_{i}+\xi_{3}P_{1i}\\ & +\xi_{4}P_{2i}+\xi_{5}B_{\text{ij}}\\ & =\xi_{6}G_{i}T_{\text{ij}}+\xi_{7}P_{1i}T_{\text{ij}}+\xi_{8}P_{2i}T_{\text{ij}}+\xi_{9}B_{\text{ij}}T_{\text{ij}}\text{,} \end{array}$$

where *G*_*i*_ is a gender indicator, *P*_1*i*_ and *P*_2*i*_ are dummy variables for place of residence corresponding to rural and urban areas and using semi-urban areas as a reference. *T*_*ij*_ is the time point at which the *j*^*th*^ measurement is taken for the *i*^*th*^ subject, which is centered at month six. *B*_*ij*_ denotes whether the *i*^*th*^ infant is breast fed or not at time *j*. The random intercept *b*_*i*_  *N* (0, *D*).

The Infant Growth dataset is analyzed with a simple logistic model, a beta-binomial model introducing only an overdispersion parameter, a random-effects logistic model that introduces a random-effects term to take the repeated structure of the data into account, and finally the combined model, which allows for both an overdispersion and a random effects term. Parameter estimates are presented in Table 2.

**Table 2:** Jimma Infant Growth Study

**Effect**	**Parameter**	**Logistic**	**Beta-binomial**
		**Estimate (s.e.,*p*)**	**Estimate (s.e.,*p*)**
Intercept	*ξ*0	−1.896(0.128, 0.001)	−0.448(1.099, 0.683)
Time	*ξ*1	0.127(0.031, 0.001)	0.188(0.090, 0.037)
Gender:Male	*ξ*2	0.027(0.025, 0.294)	0.029(0.039, 0.456)
Place rural	*ξ*3	−0.602(0.029, 0.001)	−0.949(0.501, 0.058)
Place urban	*ξ*4	−0.376(0.037, 0.001)	−0.628(0.381, 0.099)
Breast feeding	*ξ*5	0.545(0.128, 0.001)	0.788(0.347, 0.023)
Slope Gender:Male	*ξ*6	−0.003(0.006, 0.602)	−0.007(0.011, 0.534)
Slope rural	*ξ*7	0.018(0.007, 0.014)	0.029(0.020, 0.161)
Slope urban	*ξ*8	0.016(0.009, 0.097)	0.026(0.022, 0.251)
Slope Breast feeding	*ξ*9	−0.133(0.031, 0.001)	*−*0.199(0.098, 0.041)
Std. dev. random intercept	$\sqrt{d_{0}}$	—	—
Std. dev. random slope	$\sqrt{d_{1}}$	—	—
Ratio	α/β	—	1.827(1.622, 0.259)
*−*2log-likelihood		41,286	41,286
**Effect**	**Parameter**	**Logistic-normal**	**Combined**
		**Estimate (s.e.,*p*)**	**Estimate (s.e.,*p*)**
Intercept	*ξ*0	−2.741(0.186, 0.001)	−2.661(0.215, 0.001)
Time	*ξ*1	0.132(0.042, 0.002)	0.147(0.049, 0.003)
Gender:Male	*ξ*2	0.010(0.054, 0.852)	0.020(0.064, 0.751)
Place rural	*ξ*3	−0.908(0.064, 0.001)	−1.058(0.082, 0.001)
Place urban	*ξ*4	−0.581(0.082, 0.001)	−0.689(0.099, 0.001)
Breast feeding	*ξ*5	0.635(0.179, 0.001)	0.764(0.209, 0.001)
Slope Gender:Male	*ξ*6	−0.003(0.010, 0.728)	−0.005(0.012, 0.660)
Slope rural	*ξ*7	−0.015(0.011, 0.167)	0.024(0.014, 0.085)
Slope urban	*ξ*8	−0.011(0.014, 0.432)	0.015(0.017, 0.377)
Slope Breast feeding	*ξ*9	−0.149(0.044, 0.001)	−0.167(0.049, 0.001)
Std. dev. random intercept	$\sqrt{d_{0}}$	1.774(0.034, 0.001)	2.107(0.088, 0.001)
Std. dev. random slope	$\sqrt{d_{1}}$	0.193(0.007, 0.001)	0.237(0.014, 0.001)
Ratio	α/β	—	0.234(0.045, 0.001)
−2log-likelihood		37,000	36,971

Parameter estimates, standard errors, and p-values for the regression coefficients in (1) the logistic model, (2) the beta-binomial model, (3) the logistic-normal model, and (4) the combined model. Estimation was done by maximum likelihood using numerical integration over the normal random effect, if present.

Clearly, the logistic-normal model is an important improvement, in terms of likelihood, relative to both the ordinary logistic model and the beta-binomial. Moreover, considering the combined model, there is a very strong improvement in fit when the beta and normal random effects are simultaneously allowed for. The overdispesion term in the combined model is significant (p < 0.001), implying the presence of considerable extra variability due to the grouped nature of the data, which is beyond what can be accommodated by the commonly used logistic-normal model.

The logistic-normal model ignores the overdispersion that results from the grouped nature of the data. On the other hand, the beta-binomial model accommodates overdispersion which is assumed independent, implying independence between repeated measurements. Again, this is not realistic and therefore the combined model is the more viable candidate, supported further by the aforementioned 9 likelihood comparison.

The combined suggests that the intercept, the time effect, main effects of place of residence and breastfeeding are significant, which is also true for time interaction with rural place of residence and breast feeding. However, main effect and slope of gender were not significant implying that proportion of overweight seems to be invariant among male and female infants over time. Infants living in rural, and urban areas are at lower risk of overweight as compared to those in semi-urban ares with (ξ_3_ = −1.058, p = 0.001), and (ξ_4_ = −0.689, p = 0.001), respectively. Further, early initiation of breastfeeding has a protective effect against the risk of overweight in late infancy (ξ_9_ = −0.167, p = 0.001), as shown in Table 2.

### Jimma longitudinal family survey of youth

We will now analyze current school attendance. For the logit, consider the model: Y_*ij*_ |b_*i*_  Bernoulli(π_*ij*_), with

(14) $$\begin{array}{ll} \text{logit}\left(\pi_{\text{ij}}\right) & =\xi_{0}+b_{i}+\xi_{1}A_{\text{ij}}+\xi_{2}G_{i}+\xi_{3}P_{1ij}\\ & +\xi_{4}P_{2ij}+\xi_{5}W_{\text{ij}}+\xi_{6}R_{\text{ij}}\text{,} \end{array}$$

where *A*_*ij*_ is the age of the *i*^*th*^ subject at the *j*^*th*^ visit, G_i_ is the gender of the *i*^*th*^ subject. *P*_1*ij*_ and *P*_2*ij*_ denote the two dummy variables for place of residence of the *i*^*th*^ subject on the *j*^*th*^ visit, which are urban, semi-urban, and rural by taking rural as a reference. *W*_*ij*_ indicates whether the *i*^*th*^ adolescent is engaged in some work for the family or help support on the *j*^*th*^ visit. Finally, *R*_*ij*_ is the *j*^*th*^ round or measurement occasion of the *i*^*th*^ subject, and *b*_*i*_  *N* (0, *d*).

Results from fitting all four models (with/without normal random effect; with/without beta random effect) can be found in Table 3. Likelihood comparison of the beta-binomial with the standard logistic model shows no improvement in fit, implying absence of strong evidence for overdispersion. This can be noted from likelihood comparisons of the simple logistic and the beta-binomial on the one hand, as well as the logistic-normal and the combined, on the other. One can easily see, however, that the commonly used logistic-normal and the combined models are significant improvements over the standard logistic model. We further observe, while the logistic- normal model suggests a significant intercept (p = 0.045), that the same does not emerge when the combined model is considered (p = 0.099) implying the beta random effect still has some impact on the p-values. The logistic- normal model is adequate, in this case study, for the combined model where there is no strong evidence of overdispersion, as the overdispersion term is not significant (p = 0.29)for these data with two repeated measurements per subject, as mentioned in the earlier sections. Further extension by adding random slope did not improve the fit of both logistic-normal and the combined models (details not shown).

**Table 3:** Jimma Longitudinal Family Survey of Youth

**Effect**	**Parameter**	**Logistic**	**Beta-binomial**
		**Estimate (s.e.,*p*)**	**Estimate (s.e.,*p*)**
Intercept	*ξ*0	1.171(0.626, 0.061)	1.155(0.702, 0.099)
Age	*ξ*1	0.039(0.049, 0.414)	0.044(0.055, 0.421)
Place urban	*ξ*2	0.971(0.148, 0.001)	1.089(0.266, 0.001)
Place semi-urban	*ξ*3	0.979(0.159, 0.001)	1.104(0.284, 0.001)
Gender:Female	*ξ*4	*−*1.111(0.123, 0.001)	*−*1.226(0.237, 0.001)
Work	*ξ*5	0.134(0.122, 0.274)	0.146(0.138, 0.288)
Round	*ξ*6	0.341(0.141, 0.016)	0.390(0.178, 0.029)
Std. dev. random effect	$\sqrt{d}$	—	—
Ratio	*α / β*	—	0.009(0.014, 0.528)
*−*2log-likelihood		1987.7	1987.4
**Effect**	**Parameter**	**Logistic-normal**	**Combined**
		**Estimate (s.e.,*p*)**	**Estimate (s.e.,*p*)**
Intercept	*ξ*0	1.443(0.719, 0.045)	1.463(0.888, 0.099)
Age	*ξ*1	0.046(0.056, 0.408)	0.058(0.070, 0.408)
Place urban	*ξ*2	1.098(0.178, 0.001)	1.379(0.393, 0.001)
Place semi-urban	*ξ*3	1.092(0.189, 0.001)	1.339(0.368, 0.001)
Gender:Female	*ξ*4	*−*1.241(0.147, 0.001)	*−*1.499(0.339, 0.001)
Work	*ξ*5	0.153(0.144, 0.287)	0.189(0.182, 0.296)
Round	*ξ*6	0.398(0.155, 0.010)	0.519(0.237, 0.028)
Std. dev. random effect	$\sqrt{d}$	1.138(0.188, 0.001)	1.342(0.318, 0.001)
Ratio	*α / β*	—	0.013(0.013, 0.293)
−2log-likelihood		1972.9	1972.1

Parameter estimates, standard errors, and p-values for the regression coefficients in (1) the logistic model, (2) the beta-binomial model, (3) the logistic-normal model, and (4) the combined model.

Estimation was done by maximum likelihood using numerical integration over the normal random effect, if present.

Adolescents living in urban, and semi-urban areas have higher school attendance than those living in rural areas, with (ξ_2_ = 1.098, p = 0.001), and (ξ_3_ = 1.092, p = 0.001), respectively. Gender is also significantly associated with school attendance, where female adolescents are lower (ξ_4_ = −1.241, p = 0.001). There is evidence that school attendance increases in the second round visit than that of the first (ξ_6_ = 0.398, p = 0.010).

### Comparison between estimation methods

For comparison with the previously applied estimation method in the likelihood framework, we again apply the same models to the two surveys, but now in the Bayesian framework. After generating 70,000 MCMC samples for the combined, and 50,000 MCMC samples for logistic-normal, beta- binomial, and simple logistic, the first 10,000 samples are discarded and treated as so-called burn-in samples. The remaining samples are used to summarize the posterior estimates. Two distinct chains were used to check sensitivity to the initial values, and convergence was met. Convergence was checked using the Gelman-Rubin diagnostic as well as by visual inspection of the trace and QQ plots [24].

The posterior summaries of logistic, beta-binomial, logistic-normal, and combined models are given in Tables 4 and 5 for the Jimma Infants Growth dataset and the Jimma Longitudinal Family Survey of Youth, respectively. The parameter estimates are fairly similar with what was obtained previously in the likelihood approach in both cases, except for differences in the case of the beta-binomial for the Jimma Infants data in Table 4 when compared with Table 2.

**Table 4:** Jimma Infant Growth Study

**Effect**		**Logistic**	**Beta-binomial**
		**Mean(s.d.)**	**Mean(s.d.)**
Intercept	*ξ*0	−1.894(0.123)	−1.486(1.488)
Time	*ξ*1	0.126(0.031)	0.155(0.207)
Gender:Male	*ξ*2	0.027(0.026)	0.003(0.066)
Place rural	*ξ*3	−0.602(0.029)	−2.486(1.290)
Place urban	*ξ*4	−0.377(0.037)	−1.973(1.210)
Breast feeding	*ξ*5	0.543(0.123)	1.126(0.294)
Slope Gender:Male	*ξ*6	−0.003(0.006)	−0.015(0.016)
Slope rural	*ξ*7	0.018(0.007)	0.160(0.178)
Slope urban	*ξ*8	0.015(0.009)	0.1610.182)
Slope Breast feeding	*ξ*9	−0.132(0.030)	−0.289(0.097)
Std. dev. random intercept	$\sqrt{d_{0}}$	—	—
Std. dev. random slope	$\sqrt{d_{1}}$	—	—
Ratio	*α / β*	—	3.222(0.524)
*DIC*		41,310.0	40,390.0
*pD*		9.9	2511.0
**Effect**		**Logistic-normal**	**Combined**
		**Mean(s.d.)**	**Mean(s.d.)**
Intercept	ξ_0_	−2.773(0.191)	−2.755(0.258)
Time	ξ_1_	0.137(0.042)	0.169(0.062)
Gender:Male	ξ_2_	0.020(0.054)	0.026(0.069)
Place rural	ξ_3_	−0.915(0.065)	−1.115(0.085)
Place urban	ξ_4_	−0.606(0.083)	−0.749(0.103)
Breastfeeding	ξ_5_	0.666(0.185)	0.903(0.253)
Slope Gender:Male	ξ_6_	−0.003(0.010)	−0.006(0.012)
Slope rural	ξ_7_	0.015(0.011)	0.026(0.015)
Slope urban	ξ_8_	0.011(0.014)	0.017(0.018)
Slope Breastfeeding	ξ_9_	−0.144(0.041)	−0.192(0.061)
Std. dev. random intercept	$\sqrt{d_{0}}$	1.783(0.035)	2.212(0.074)
Std. dev. random slope	$\sqrt{d_{1}}$	0.193(0.007)	0.250(0.013)
Ratio	*α / β*	—	0.288(0.031)
*DIC*		33,605.1	33,377.6
*pD*		5400.7	6218.3

Estimated posterior mean and standard deviation in (1) the logistic model, (2) the beta-binomial model, (3) the logistic-normal model, and (4) the combined model.

**Table 5:** Jimma Longitudinal Family Survey of Youth

**Effect**		**Logistic**	**Beta-binomial**
		**Mean(s.d.)**	**Mean(s.d.)**
Intercept	ξ_0_	1.185(0.624)	1.151(0.731)
Age	ξ_1_	0.039(0.049)	0.047(0.057)
Place urban	ξ_2_	0.977(0.148)	1.134(0.183)
Place semi-urban	ξ_3_	0.987(0.161)	1.161(0.202)
Gender:Female	ξ_4_	−1.113(0.123)	−1.266(0.148)
Work	ξ_5_	0.133(0.122)	0.154(0.140)
Round	ξ_6_	0.343(0.142)	0.404(0.165)
Std. dev. random effect	$\sqrt{d}$	*—*	*—*
Ratio	*α / β*	*—*	0.0111(0.0029)
*DIC*		2002.0	2001.0
*pD*		6.97	13.77
**Effect**		**Logistic-normal**	**Combined**
		**Mean(s.d.)**	**Mean(s.d.)**
Intercept	ξ_0_	1.452(0.732)	1.272(0.953)
Age	ξ_1_	0.047(0.057)	0.077(0.078)
Place urban	ξ_2_	1.107(0.180)	1.427(0.270)
Place semi-urban	ξ_3_	1.104(0.192)	1.382(0.269)
Gender:Female	ξ_4_	−1.247(0.149)	−1.528(0.214)
Work	ξ_5_	0.155(0.145)	0.199(0.184)
Round	ξ_6_	0.401(0.157)	0.521(0.203)
Std. dev. random effect	$\sqrt{d}$	1.148(0.203)	1.417(0.266)
Ratio	*α / β*	—	0.013(0.003)
*DIC*		1943.0	1915.0
*pD*		241.5	211.9

Estimated posterior mean and standard deviation in (1) the logistic model, (2) the beta-binomial model, (3) the logistic-normal model, and (4) the combined model.

In terms of significance of the parameters, the same conclusion is reached for the two case studies in both approaches, except that the beta-binomial for the intercept and time effects in Jimma infants study shows significance in the likelihood framework as given in Section (The jimma infant growth study), while the same does not emerge from the Bayesian case, as observed from the 95% credible inteval which include zero for these effects. We compared the various models using the DIC criterion. For both studies, there is a significant reduction in the DIC of the logistic-normal and the beta-binomial, as compared to the simple logistic. We observe a rather high degree of model improvement by combining beta and normal random effects simultaneously, to allow for both the overdispersion and the data hierarchy. Moreover, the logistic and the beta-binomial ignore the correlation stemming from the data hierarchy on the one hand, and the logistic-normal does not allow for the overdispersion, on the other, which altogether make the combined model the preferred one.

According to Spiegelhalter et al [22], in comparing complex hierarchical models where the number of parameters are not clearly defined, *pD* is the difference between the posterior mean of the deviance and the deviance at the posterior means of the parameters of interest, not only measures the effective number of parameters but also the model complexity. These authors further noted that the contribution *pD*_*i*_ of each observation *i* turned out its leverage, defined as the relative influence that each observation has on its own fitted value. for *y*_*i*_ conditionally independent given θ, *pD*_*i*_, shows its interpretation as the difficulty in estimating θ with *y*_*i*_. This shows the connection between the sample size, the parameters to be estimated, and the *pD*. The Jimma infants (n = 7969) and the Jimma Longitudinal family survey (n = 2100) data have large number of subjects followed longitudinally, where each subject was measured seven and two times, respectively. Due to these reasons, the *pD* values, as presented in Tables 4 and 5, appeared to be larger as the by-product of the MCMC estimation to obtain leverage of each observation.

Unlike the Jimma infants study in Table 4, *pD* of the combined model for the Jimma Longitudinal Family Survey of Youth in Table 5, (*pD* = 211.9), is lower than that of the logistic-normal (*pD* = 241.5). This implies that, for the Jimma Longitudinal Family Survey of Youth, the combined model is less complex to fit than the logistic-normal, although, this is what we don’t usually expect, as the combined model seems more complex, since it includes both the beta and the normal random effects, while, the logistic-normal including only the normal random-effects. However, for these specific data, this resulted likely because there is less conflict between the specific data set, and the prior distributions which could be associated to the conjugacy of the beta random effects, as well as the peculiar data features including number of subjects and repeated measurements per subject.

The SAS and WinBugs codes used for analysis of the data sets are given in the Appendix.

## Discussion

Analysis of the case studies show that, in the presence of overdispersion, and clustering, the combined model results in improvement in model fit, which is similar to the finding in Molenberghs et al [9].

This study revealed that early breastfeeding lowers the risk of overweight at late infancy. This finding is in line with Bergman et al [10], who showed that breastfed infants had lower BMI after 3 months from birth than bottle-fed infants, though the BMIs at birth were nearly identical in both groups. Owen et al [11], who reviewed sixty-one studies, states that initial breastfeeding protects against obesity in later life, although the precise magnitude of the association remains unclear. Unlike Owen et al [11], the present study showed that, infants in the breastfed group were fatter, at birth, as compared to those who were not breastfed. This is likely because of the unmeasured maternal history, such as maternal BMI, and socio-cultural aspects, which are considered to be the risk factors of overweight in children [25]. In addition, it is a common practice in the study area that mothers provide additional liquid or solid food starting from early infancy, in addition to breastfeeding. This is probably because they believe that a child with more weight is considered as healthy, which is likely to have its own impact on the BMI in the early infancy. In this study, it is also shown that place of residence does not have a long term effect in the risk of overweight, instead it is the mode of feeding which is more important. The baseline differences observed in the risk of overweight among infants living in urban, semi-urban areas might be attributable to other family related factors like, social class, family income, educational level of the parents, and other socio-cultural variables, which are indicated to affect the nutrition of young children and women in Ethiopia [12].

In investigating school attendance among adolescents, this study showed that, girls have a lower rate of current school attendance than boys, which is a common situation in most Sub-Saharan African Countries. According to World Health Organization [13], there is a clear gender gap observed in primary or secondary school enrollment when the Gender Parity Index (GPI), the ratio of female to male enrollment, is considered. Between the years 1999 and 2003, GPI was found to be 0.7, indicating that there were only 7 girls enrolled at primary schools for every 10 boys. This gender gap increases with increasing level of education. This study also showed that adolescents in urban and semi-urban areas have higher rate of than those in the rural areas, which is in line with report of World Bank [14], where it was stated that among children in rural areas with a school in the neighborhood, less than 44% registered for school; in urban areas, the percentage is much higher up to 86%. According to the report, distance to the nearest school, household characteristics, and learning environment were among the possible reasons of the gap in the school attendance.

## Conclusion

We have presented a model which integrates normal and beta random effects into a single model, termed the combined model. Our work builds upon that of Molenberghs et al [9], who brought together normal random effects to induce association between repeated binary and binomial data, and a beta-binomial distributed random factor in the log-linear predictor to fine tune the overdispersion.

Maximum likelihood estimation was considered by integrating over the random effects using the SAS procedure NLMIXED.

Further, Bayesian inference has been applied. Prior information about the parameters induces correlation, which then leads to reduced effective dimensionality although the reduction depends on the available data [22]. Complexity reflects the difficulty in fit and hence it seems reasonable that the measure of complexity may depend on both the prior information concerning the parameters under scrutiny and the specific data that are observed. This can be elucidated from the Jimma Longitudinal Family Survey of Youth result, where the combined model is less complex in fit, which likely results from the conjugacy of the beta random effect and the number of subjects as well as the repeated measurements per subject.

Future studies on early growth of children could benefit from careful measurement of a wider range of potential confounders of overweight.

Further efforts should be made to fill the gap in school attendance among boys and girls, as well as, urban and rural areas by focusing on the potential causes, such as lagging experience in primary schooling, which is then exacerbated by such factors as the practice of early marriage among Ethiopian women, families reluctance to invest in girls education. Situating schools closer to childrens homes in rural areas, and improve the quality of the services is necessary [14]. Longitudinal studies with better number of repeated measurements per subject should be conducted to get better insight on the trends of school enrollment, survival of adolescents.

## Appendix

### SAS Implementation

This section shows a SAS program, using the procedure NLMIXED, for the combined model.

#### Jimma infants growth study

proc nlmixed data = infant noad qpoints = 10;

title 'Combined Model-Jimma infants with const = beta/alpha';

parms Beta_0 = −3.23 Beta_1 = 0.0602 beta_2 = 0.0402 Beta_3 = −0.8369 Beta_4 = −0.552

Beta_5 =1.7266 Beta_6 = −0.003 Beta_7 = −0.0262 Beta_8 = −0.0184 Beta_9 = −0.1584

sd1 = 1.3662 sd2 = 0.2576 const = 0.0944;

eta = Beta_0 + b1+ (Beta_1 + b2)*time + Beta_2*sex + Beta_3*(place = 1) + Beta_4*(place = 2)

+Beta_5*(Bf) + Beta_6*(sex)*time + Beta_7*time*(place = 1) + Beta_8*time*(place = 2)

+ Beta_9*time*(BF);

expeta = exp(eta);

ll = −log(1 + const) + BMIBIN*eta - BMIBIN*log(1 + expeta)

+ (1-BMIBIN)*log((1-expeta/(1 + expeta)) + const);

model BMIBIN ~ general(ll);

random b1 b2 ~ normal([0,0],[sd1**2,0,sd2**2]) subject = id;

run;

#### **The Jimma Longitudinal Family Survey of Youth**

proc nlmixed data = ado noad qpoints = 10 ;

title 'Combined Model-Jimma youth with const = beta/alpha';

title3 'Retriction beta/alpha = const';

parms Beta_0 =1.1652 Beta_1 = 0.04351 Beta_2 = 1.0911 Beta_3 = 1.1051

Beta_5 = −1.2249 Beta_6 = 0.1471 Beta_7 = 0.3903 const = 0.05 sd = 0.5;

eta = Beta_0 + Beta_1*age + Beta_2*(typplace = 1)

+ Beta_3*(typplace = 2) + Beta_5*currwork + Beta_6*sex

+Beta_7*round + b1;

expeta = exp(eta);

ll = −log(1 + const) + currscho*eta - currscho*log(1 + expeta)

+ (1-currscho)*log((1-expeta/(1 + expeta)) + const);

model currscho ~ general(ll);

random b1 ~ normal(0,sd*sd) subject = id ;

run;

### **WinBugs Implementation**

This section presents a WinBugs program for the combined model.

#### **Jimma infants growth study**

model {

for (i in 1:49112) {

BMIBIN[i] ~ dbern(p[i])

p[i] < −kappa[i]*theta[i]

theta[i] ~ dbeta(a,b)

logit(kappa[i]) < − alpha0 + (s[ID[i]] + alpha1)*TIME[i]

+alpha2*SEX[i] + alpha3*RUR[i] + alpha4*URB[i] + alpha5*BF[i]

+alpha6 * SEX[i]*TIME[i] + alpha7 * RUR[i] *TIME[i]

+ alpha8*URB[i]*TIME[i] + alpha9*BF[i]*TIME[i]

+ u[ID[i]]

}

for (j in 1:7969) {

u[j] ~ dnorm(0.0,tau1)

s[j] ~ dnorm(0.0,tau2)

}

a ~ dunif(3,5)

b ~ dunif(1.1,1.5)

c < −b/a

alpha0 ~ dnorm(0.0,1.0E-6)

alpha1 ~ dnorm(0.0,1.0E-6)

alpha2 ~ dnorm(0.0,1.0E-6)

alpha3 ~ dnorm(0.0,1.0E-6)

alpha4 ~ dnorm(0.0,1.0E-6)

alpha5 ~ dnorm(0.0,1.0E-6)

alpha6 ~ dnorm(0.0,1.0E-6)

alpha7 ~ dnorm(0.0,1.0E-6)

alpha8 ~ dnorm(0.0,1.0E-6)

alpha9 ~ dnorm(0.0,1.0E-6)

tau1 ~ dgamma(0.001,0.001)

tau2 ~ dgamma(0.001,0.001)

sd1 < −sqrt(1/tau1)

sd2 < −sqrt(1/tau2)

}

#### **The Jimma Longitudinal Family Survey of Youth**

Model {

for (i in 1:3815) {

SCHO[i] ~ dbern(p[i])

p[i] < −theta[i]*kappa[i]

theta[i] ~ dbeta(a,b)

logit(kappa[i]) < − alpha0 + alpha1*AGE[i] + alpha2*URB[i]

+alpha3*SURB[i] + alpha4*WORK[i] + alpha5 * SEX[i]

+ alpha6 * ROUND[i] + u[ID[i]]

}

for (j in 1:1956) {

u[j] ~ dnorm(0,tau)

}

a ~ dunif(110,210)

b ~ dunif(1.1,2.2)

c < −b/a

alpha0 ~ dnorm(0.0,1.0E-6)

alpha1 ~ dnorm(0.0,1.0E-6)

alpha2 ~ dnorm(0.0,1.0E-6)

alpha3 ~ dnorm(0.0,1.0E-6)

alpha4 ~ dnorm(0.0,1.0E-6)

alpha5 ~ dnorm(0.0,1.0E-6)

alpha6 ~ dnorm(0.0,1.0E-6)

tau ~ dgamma(0.001,0.001)

sd < −1/sqrt(tau)

}

## Competing interests

The authors declare that they have no competing interests.

## Authors’ contribution

The first two authors have done the programming of the statistical methodology and wrote the first draft of the paper. The two last authors contributed to the statistical methodology and finalization of the writing. All authors read and approved the final manuscript.
